# Bibliography

**Published:** 1918-06

**Authors:** 


### BIBLIOGRAPHY

*General Pathology and Bacteriology for Dental Students.* This is the second edition of McConnell's pathology. It is a concise and well written book by Guthrie McConnell, M.D., who for several years taught this subject in the Philadelphia Dental College. It is very well written and fully illustrated with well executed cuts of the pathologic conditions of the body tissues. It would have a more useful field of service in presenting this difficult subject if more attention were given to the strictly dental phases of disease. We would suggest that the next edition could be made more interesting by collaborating with a dentist who has had a wider experience in the study of the more strictly dental features of disease. This should not detract from the scientific character of the work and would certainly add interest at least to the student's grasp of this somewhat complicated subject. We realize that strictly speaking pathology is the same in principle in all parts of the body, but its application to dental and oral structures in an accentuated manner is needed to claim the close study required to master its details, which are somewhat tiresome unless pointed by interesting illustrative material. The book is all that anyone could desire from the publisher's standpoint, and it should be a most valuable addition to dental literature and facilitate a better knowledge of mouth diseases. It is published by the W. B. Saunders Co., of Philadelphia. $2.50 net.

---

*Electrolytic Medication.* Is a little booklet put out by the Ritter Dental Manufacturing Co., to illustrate and describe the appliances developed to care for the problem of ionization of the root environment. It contains a brief but clear statement of the theory and practice of this treatment with illustrations of the necessary facilities for producing the treatment with accuracy and safety. It is a handy book to possess and every one should have a copy whether he engages in this practice or not as it is very instructive. A copy can be obtained directly from the Ritter Co., or from the dental depots.

---

*Dental Electro-Therapeutics.* Lea and Febiger of Philadelphia has just issued the second edition of this work as edited by Ernest Sturridge, L.D.S., D.D.S., of London, England. In this book the author has given us a very terse and satisfactory statement of the science involved in applied radiography and electro-therapeutics to dental practice. The book is well illustrated and the text is admirably clear and logically arranged. In this day of applied electrolysis to the difficulties of medicating apical and root environments directly, it has become necessary for every dentist to have an adequate knowledge of the technique involved in this form of treatment. This little book of 300 pages sets out the fundamental details involved better than anything we have seen, especially for therapeutic treatment. The radiographic part of the book is not so amply treated as the therapeutic, but it gives briefly the essentials of the art of radiographic diagnosis. The live interest at present in the subject of better root-canal work ought to bring a ready sale for this book. We commend it cordially for the valuable information which it presents in a concise and scientific manner. The book may be had from the publishers or from the dental depots.

---

*Elementary Dental Radiography.* The second edition of Dr. Howard R. Raper's book on radiography is just published by the Consolidated Dental Manufacturing Co. This is a large book of about 500 pages, profusely illustrated, on the subject of Dental Radiography only. The dental profession is familiar with the excellent work done by Dr. Raper in this field. He was among the first to take up this work, and because of his enthusiasm and constant devotion he has made a reputation for thorough and accurate scientific work along this line that entitles him to write with authority on this subject. The book is filled with beautiful illustrations of every phase of radiography, from the machinery involved to the technical and therapeutic principles and practice of the art. No other work is so complete, and the author has certainly put the dental profession under tremendous obligations for his hard labors and the able book he has made for us. No one who practices radiography, or who only has to read and interpret the films, can afford to be without this book, as it is intensely practical and most comprehensive in its details. It is one of the best books ever produced on this subject. The publishers have done an excellent piece of work in its typography.

